# Nrf2^−/−^ regulated lung DNA demethylation and *CYP2E1* DNA methylation under PM_2.5_ exposure

**DOI:** 10.3389/fgene.2023.1144903

**Published:** 2023-03-20

**Authors:** Mengjie Wu, Menghui Jiang, Hao Ding, Siying Tang, Daochuan Li, Jingbo Pi, Rong Zhang, Wen Chen, Rui Chen, Yuxin Zheng, Jinmei Piao

**Affiliations:** ^1^ School of Public Health, Qingdao University, Qingdao, China; ^2^ The Municipal Government Hospital of Zibo, Zibo, Shandong, China; ^3^ Qingdao Chengyang District Center for Disease Control and Prevention, Qingdao, China; ^4^ Department of Toxicology, School of Public Health, Sun Yat-Sen University, Guangzhou, China; ^5^ School of Public Health, China Medical University, Shenyang, China; ^6^ Department of Toxicology, School of Public Health, Hebei Medical University, Shijiazhuang, China; ^7^ School of Public Health, Capital Medical University, Beijing, China

**Keywords:** PM2.5, Nrf2, CYP2E1, DNA methylation, Tet3

## Abstract

Cytochrome P450 (CYP450) can mediate fine particulate matter (PM_2.5_) exposure leading to lung injury. Nuclear factor E2-related factor 2 (Nrf2) can regulate CYP450 expression; however, the mechanism by which Nrf2^−/−^ (KO) regulates *CYP450* expression *via* methylation of its promoter after PM_2.5_ exposure remains unclear. Here, Nrf2^−/−^ (KO) mice and wild-type (WT) were placed in a PM_2.5_ exposure chamber (PM) or a filtered air chamber (FA) for 12 weeks using the real-ambient exposure system. The *CYP2E1* expression trends were opposite between the WT and KO mice following PM_2.5_ exposure. After exposure to PM_2.5,_ *CYP2E1* mRNA and protein levels were increased in WT mice but decreased in KO mice, and CYP1A1 expression was increased after exposure to PM_2.5_ in both WT and KO mice. CYP2S1 expression decreased after exposure to PM_2.5_ in both the WT and KO groups. We studied the effect of PM_2.5_ exposure on *CYP450* promoter methylation and global methylation levels in WT and KO mice. In WT and KO mice in the PM_2.5_ exposure chamber, among the methylation sites examined in the *CYP2E1* promoter, the CpG2 methylation level showed an opposite trend with CYP2E1 mRNA expression. The same relationship was evident between CpG3 unit methylation in the *CYP1A1* promoter and CYP1A1 mRNA expression, and between CpG1 unit methylation in the *CYP2S1* promoter and CYP2S1 mRNA expression. This data suggests that methylation of these CpG units regulates the expression of the corresponding gene. After exposure to PM_2.5_, the expression of the DNA methylation markers ten-eleven translocation 3 (TET3) and 5-hydroxymethylcytosine (5hmC) was decreased in the WT group but significantly increased in the KO group. In summary, the changes in CYP2E1, CYP1A1, and CYP2S1 expression in the PM_2.5_ exposure chamber of WT and Nrf2^−/−^ mice might be related to the specific methylation patterns in their promoter CpG units. After exposure to PM_2.5,_ Nrf2 might regulate CYP2E1 expression by affecting CpG2 unit methylation and induce DNA demethylation *via* TET3 expression. Our study revealed the underlying mechanism for Nrf2 to regulate epigenetics after lung exposure to PM_2.5_.

## 1 Introduction

Atmospheric pollution levels have improved throughout China; However, air pollution in Shijiazhuang, Hebei, located in northern China, is still serious in the seasonal heating season during winter. PM_2.5_ (aerodynamic diameter <2.5 µm) is the main component of air pollution that cause lung damage and affect lung function in organisms during short-term exposure (Li D. et al., 2019; Jiang et al., 2021; Strassmann et al., 2021). Previous studies have found that the expression of the CYP450 enzyme in lung tissue is related to the change in lung function after exposure to PM_2.5_ (Kim et al., 2018).

Exogenous chemicals in PM_2.5_ can be metabolized by cytochrome P450 (CYP450) enzymes, resulting in lung cell damage (Billet et al., 2007). CYP2E1, CYP1A1, and CYP2S1 are the key metabolic enzymes in phase I and are mainly located in the membrane of the endoplasmic reticulum (ER) and mitochondrion (Mittal et al., 2015; Popescu et al., 2021). PM_2.5_ exposure increases the expression of CYP2E1, which leads to lung injury *via* endoplasmic reticulum stress (Ding et al., 2021). Exogenous chemicals in PM_2.5_ can be metabolized by CYP1A1 to induce oxidative stress and inflammation, which lead to human lung cell injury (Abbas et al., 2019). CYP2S1 metabolizes various exogenous chemicals, such as PAHs, in atmospheric fine particulate matter (Al Zallouha et al., 2020), dioxins and naphthalene are toxic and potentially carcinogenic PAHs (Karlgren et al., 2005) that may be key players in lung injury.

Nuclear factor erythroid 2-related factor 2 (Nrf2) is a transcriptional factor of the bZIP family and is also a core transcription factor in anti-oxidative stress, regulating multiple antioxidant genes (Tebay et al., 2015). Our previous study has shown that PM_2.5_ exposure reduced lung function in wild-type (WT) mice; However, compared with a filtered air group, PM_2.5_ exposure has no obvious effect on the lung function and pathology of Nrf2^−/−^ (KO) mice. Changes in CYP450 expression in KO mice following PM_2.5_ exposure, thereby affecting endoplasmic reticulum stress, which is closely related to lung injury (Ding et al., 2021). Numerous studies have illustrated that Nrf2 can regulate the expression of CYP450 (Wu et al., 2012; Ashino et al., 2020).

Gene expression is regulated by DNA methylation, and the hypomethylation of CpG islands in gene promoters is associated with gene activation (Edgar et al., 2014); However, promoter CpG island hypermethylation represses gene expression (Rider and Carlsten, 2019). The homeostasis between DNA methylation and demethylation is a crucial mechanism that protects the stability of organisms (Robertson, 2005). DNA methyltransferases (DNMTs) are responsible for DNA methylation, which transfers the methyl group of S-adenosyl methionine (SAM) to cytosine in DNA (Lyko, 2018). Gene silencing is caused by gene promoter hypermethylation (Moore et al., 2013; Fukui et al., 2019). DNA demethylation involves a ten-eleven translocation methylcytosine dioxygenase (TETs) to oxidize 5-methylcytosine (5 mC) into 5-hydroxymethylcytosine (5hmC) (Kafer et al., 2016). DNA methylation affects *CYP450* expression (Liu et al., 2022). In a study of lung injury caused by smoking, DNA methylation was found to be the main regulator of CYP450 enzyme expression (Tekpli et al., 2012). In addition, the previous study found that PM_2.5_ exposure causes lung injury and is associated with DNA methylation changes (Shi et al., 2019). However, it is unclear whether CYP450 enzyme gene expression is affected by DNA methylation in Nrf2^−/−^ mice exposed to PM_2.5_.

A real-ambient exposure system was used in the present study, which realistically simulated the surrounding atmospheric environment. An independent ventilation cage system equipped with or without three layers of high-efficiency particulate air filters that can filter particulate matter in filtered air chambers and not filter in PM_2.5_ exposure chambers was constructed in Shijiazhuang, Hebei Province (Jiang et al., 2020; Jiang et al., 2021). The system accurately simulated real air pollution in the environment (Tang et al., 2022).

In our study, Nrf2^−/−^ mice exposed to PM_2.5_ exhibited decreased CYP2E1 expression in the lung, potentially due to the regulatory effect of Nrf2 on CYP2E1 expression *via* CpG unit methylation. Nrf2 may regulate DNA demethylation by affecting TET3 expression after PM_2.5_ exposure. Our study provides a new theoretical basis by which Nrf2 regulates epigenetics after PM_2.5_ exposure.

## 2 Materials and methods

### 2.1 Animal and real-ambient exposure system

The Nrf2^−/−^ mice were modeled by Professor Masayuki Yamamoto at Tohoku University and provided by Jingbo Pi Laboratory, China Medical University (Jiang et al., 2021). And Nrf2^+/−^ mice were bred in male and female to obtain Nrf2^−/−^ mice and WT was Nrf2^+/+^ mice, and PCR genotyping was used to distinguish them (Ding et al., 2021; Jiang et al., 2021). The real-ambient exposure system in this study is located in Shijiazhuang, and the exposure device is as described in the previous study (Li D. et al., 2019; Jiang et al., 2021). In short, the IVC exposure system consists of a control and exposure chamber, which is connected to a three-layer HEPA filter to produce filtered air (FA), and an exposure chamber which is connected to unfiltered air (PM). The factors (temperature, airflow, humidity, pressure, ventilation frequency, air flow rate, and noise) in both chambers are consistent (Ding et al., 2021). Eight-week-old Nrf2 Knockout mice and WT mice were placed in FA and PM chambers (10 mice per group), and the mice were given food and water freely in a circulating chamber with 12 h light and 12 dark. Mice were exposed for 16 h daily and 7 days/week for 12 weeks (Jiang et al., 2021). We analyzed particle size profiles in both chambers with an Aerodynamic Particle Sizer Spectrometer 3321 and measured PM_2.5_ concentrations with the Aerosol Detector DUSTTRAKTM II (TSI Incorporated, Shoreview, MN, United States). Our previous study showed that PM_2.5_ exposure concentration exceeded 35 μg/m^3^ on 76 of 12 weeks of exposure (84 days) and exceeded 150 μg/m^3^ on 28 days (Li D. et al., 2019). The average daily concentration of PM_2.5_ in the ambient air studied was 130.22 μg/m^3^, and the average daily concentration of PM_2.5_ in the exposed room was 85.24 μg/m^3^ (Li D. et al., 2019). Based on our previous methods, the cumulative pulmonary exposure to PM_2.5_ was calculated to be 154.79 μg (Jiang et al., 2021). The ethical committees of Hebei Medical University (IACUC-Hebmu-20170116) approved the animal experiments and complied with all animal ethics regulations.

### 2.2 Quantitative real-time PCR

Extraction of Total mRNA from mice lung tissue using Trizol reagent (Thermo Science, Waltham, United States). We used reverse transcription kits for the synthesis of cDNA (Takara, Kyoto, Japan). SYBR Green PCR Master Mix was used for real-time quantitative PCR (qRT-PCR) (Thermo Fisher Science, Waltham, United States). The expression of DNA damage response enzymes was measured by qRT-PCR. The target gene was compared with *ß*-actin, and the relative expression was calculated. The primers used are described in Supplemental Material Table S1.

### 2.3 Western blot

Mice lung tissue was homogenized with a mixture containing phenylmethylsulfonyl fluoride (PMSF), alkaline phosphatase inhibitors, and protease inhibitors (Solarbio, Beijing). Protein concentrations were determined with the BCA protein analysis kit (Solarbio, Beijing, China) according to its product instructions. Using a 10% (SDS-PAGE) gel, transfer the protein to a polyvinylidene fluoride (PVDF) membrane (MILLIBOLE, Billerica, MA). Block with skim milk powder for 2 hours at normal temperature, and incubated overnight at 4°C with primary antibodies including CYP2E1 (Affinity, Beijing, 1:1,000), Nrf2 (Cell Signaling Technology, Boston, 1:1,000), GAPDH (ABclonal, Wuhan 1:1000), CYP1A1 (ABclonal, Wuhan 1:1000), CYP2S1 (absin, Shanghai 1:1000), DNMT1 (ABclonal, Wuhan 1:1000), TET1 (ABclonal, Wuhan 1:1000), TET3 (ABclonal, Wuhan 1:1000) and then goat anti-rabbit IgG secondary antibody (Epizyme, Shanghai, China) was incubated at room temperature for 2 h. Detection was performed using the ECL system (Millipore, Billerica, MA, United States) and analysis was performed using ImageJ (NIH, United States) software.

### 2.4 Immunohistochemistry

Mice’s lung tissue was fixed with formalin and embedded in paraffin. Samples were cut into 5 μm sections and dewaxed in water, and antigen repair was performed. Serum block at room temperature for 30 min. The sections were incubated with CYP1A1, CYP2E1, CYP2S1, TET3, TET1, DNMT1, 5hmC and *γ*-H2AX antibodies at 4°C overnight. After washing with PBS (PH7.4), the cells were incubated with HRP-labeled secondary antibody for 50 min at room temperature, then DAB developer was added to the drop, and hematoxylin was used as the compound stain. Five regions were randomly selected from the slices and quantified by ImageJ (NIH, United States) using the research method described previously (Wong et al., 2019).

### 2.5 DNA methylation analysis by MassARRAY

We selected 5000bp upstream and 1000bp downstream of the transcription start site from the gene sequence of NCBI (GRCm39 version), finally, we selected the regions with relatively dense CpG sites for detection. Mice lung DNA was extracted with the DNA extraction kit (BioTeKe Corporation) according to the instructions, and bisulfite was modified with a NaHSO3 kit (ZYMO Research). Sequenom EpiTYPER analysis was performed following the protocol recommended by the manufacturer. EpiDesigner (Agena) was used to design the target region primers, and the PAGE primer purification method was used to synthesize the PCR primer sequences of the corresponding fragments. For each reverse primer, an additional T7 promoter tag for *in vivo* transcription was added, while a 10 M tag on the forward primer was used to regulate the unchain temperature difference Cycling conditions: 4 min at 94°C followed by 45 cycles of 94°C for the 20°s, 56°C for 20°s, and 72°C for 1min followed by 72°C for 3°min. PCR products processed according to the manufacturer’s instructions were used for MassARRAY analysis, and the relative amount of methylation can be calculated by comparing the signal intensities between the quality signals of methylated and unmethylated template DNA. Methylation ratios of individual units were generated by EpiTYPER™ (Agena, Inc.) software, inapplicable readings, and their corresponding sites were excluded from the analysis. The primer sequences are shown in Supplementary Table S2.

### 2.6 SAM and SAH detected by ELISA

SAM and SAH were measured with ELISA kits (Shanghai FANKEL Industrial Co., Ltd.). Three times were repeated for all standards and samples according to the product instructions, and the absorbance (OD) was read at 450 nm to calculate the concentration of SAM SAH in the lung.

### 2.7 Statistical analysis

Statistical analysis was performed using GraphPad Prism (8.30) software. Data are presented as mean ± SEM. Differences between control and exposed groups were assessed by two-way analysis of variance (TWO WAY-ANOVA). Results were considered statistically significant as follows: * *p* < 0.05; ** *p* < 0.01; *** *p* < 0.001; **** *p* < 0.0001.

## 3 Results

### 3.1 The antioxidant levels of WT and Nrf2^−/−^ mice decreased after PM_2.5_ exposure

In WT mice, Nrf2 downstream genes except for superoxide dismutase-1 (SOD1), which was increased after PM_2.5_ exposure, other antioxidant indexes such as superoxide dismutase-2 (SOD2), heme oxygenase-1 (HO-1), glutathione peroxidase (GSH-Px) were significantly decreased after PM_2.5_ exposure, while there was no significant difference in antioxidant levels after PM_2.5_ exposure in the Nrf2 knockout mice compared with the FA group (Supplementary Material). These results suggested that PM_2.5_ exposure reduced the antioxidant level in wild-type mice, but after Nrf2 knockout, there was no significant change in antioxidant level in the FA group and PM_2.5_ group.

### 3.2 Expression of four groups of CYP450 enzymes

**FIGURE 1:**
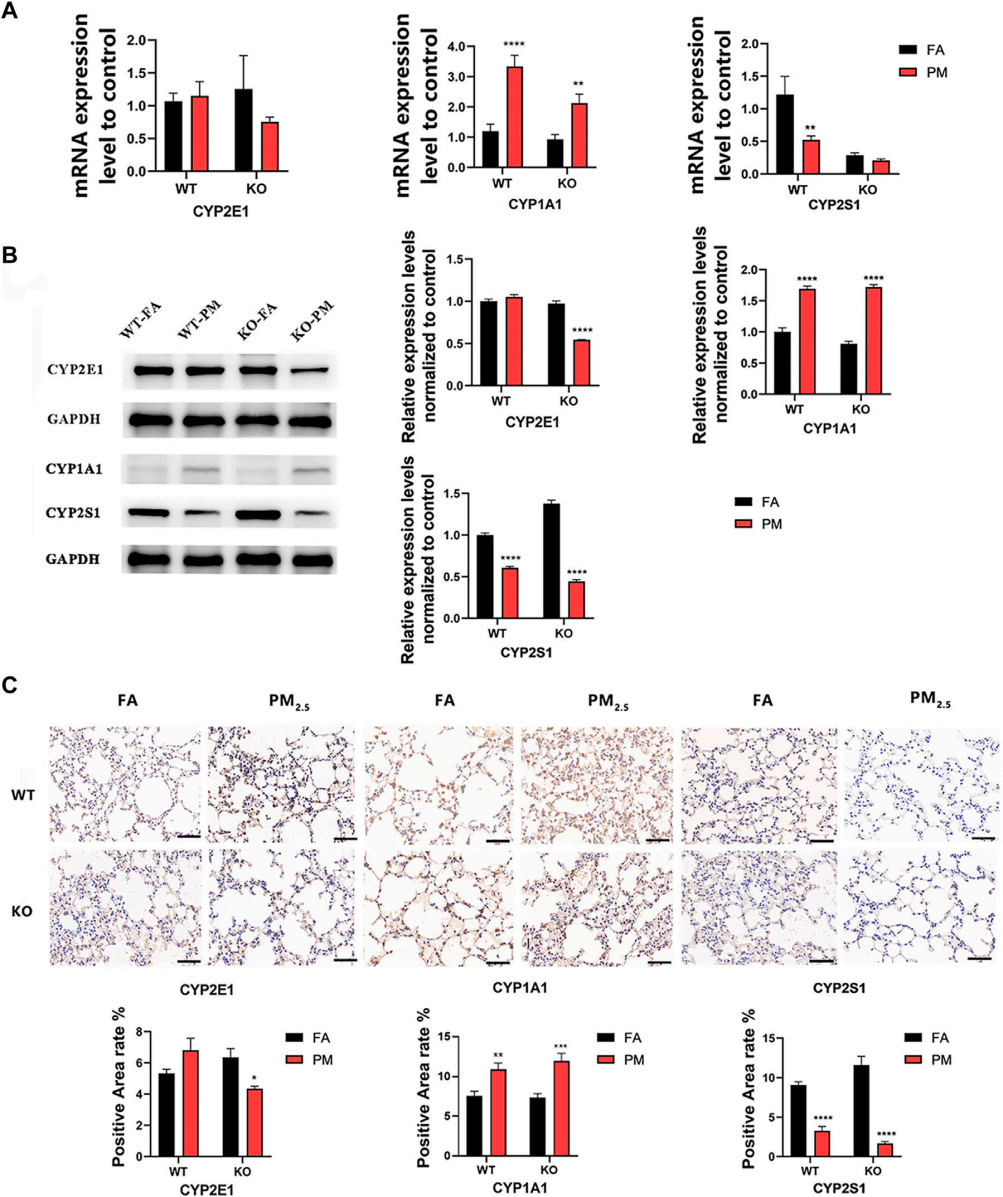
Expression levels of CYP450 enzyme in WT group and Nrf2^−/−^group. **(A)** Changes in mRNA levels of CYP450 enzyme. **(B)** The expression of CYP450 enzyme protein levels was analyzed by WB and quantified by ImageJ analysis. *n* = 3 per group. **(C)** Immunohistochemical detection of CYP450 protein expression in four groups and quantitative analysis of the positive area. *n* = 2 per group. Scale bars are 50 μm, FA, filtered air; PM, fine particulate matter. WT, wild-type mice; KO, Nrf2^−/−^ mice. Error bars represent mean ± SEM, *p < 0.05, **p < 0.01, ***p < 0.001, ****p < 0.0001.

We explored the expression of key cytochrome P450 (CYP450) enzyme genes during exposure for 12 weeks (Figure 1A). The results showed that in wild-type mice, the mRNA expression levels of CYP2E1 and CYP1A1 increased after exposure to PM_2.5_, while *CYP2S1* expression decreased. In KO mice, the *CYP1A1* expression was increased, but the expression level of *CYP2E1* and *CYP2S1* decreased after PM_2.5_ exposure. Additionally, in Nrf2^−/−^ mice exposed to PM_2.5_, the CYP2E1 protein level was significantly reduced but elevated in WT mice **(** Figures 1B, C). In WT and KO mice, CYP1A1 protein level increased after PM_2.5_ exposure, while CYP2S1 expression decreased after PM_2.5_ exposure, the trend was consistent with that of mRNA (Figures 1B, C). Overall, it suggested that PM_2.5_ exposure could affect the expression of CYP450 enzyme in WT and Nrf2^−/−^ mice.

### 3.3 Expression levels of DNA methylation-related enzymes in four groups

**FIGURE 2:**
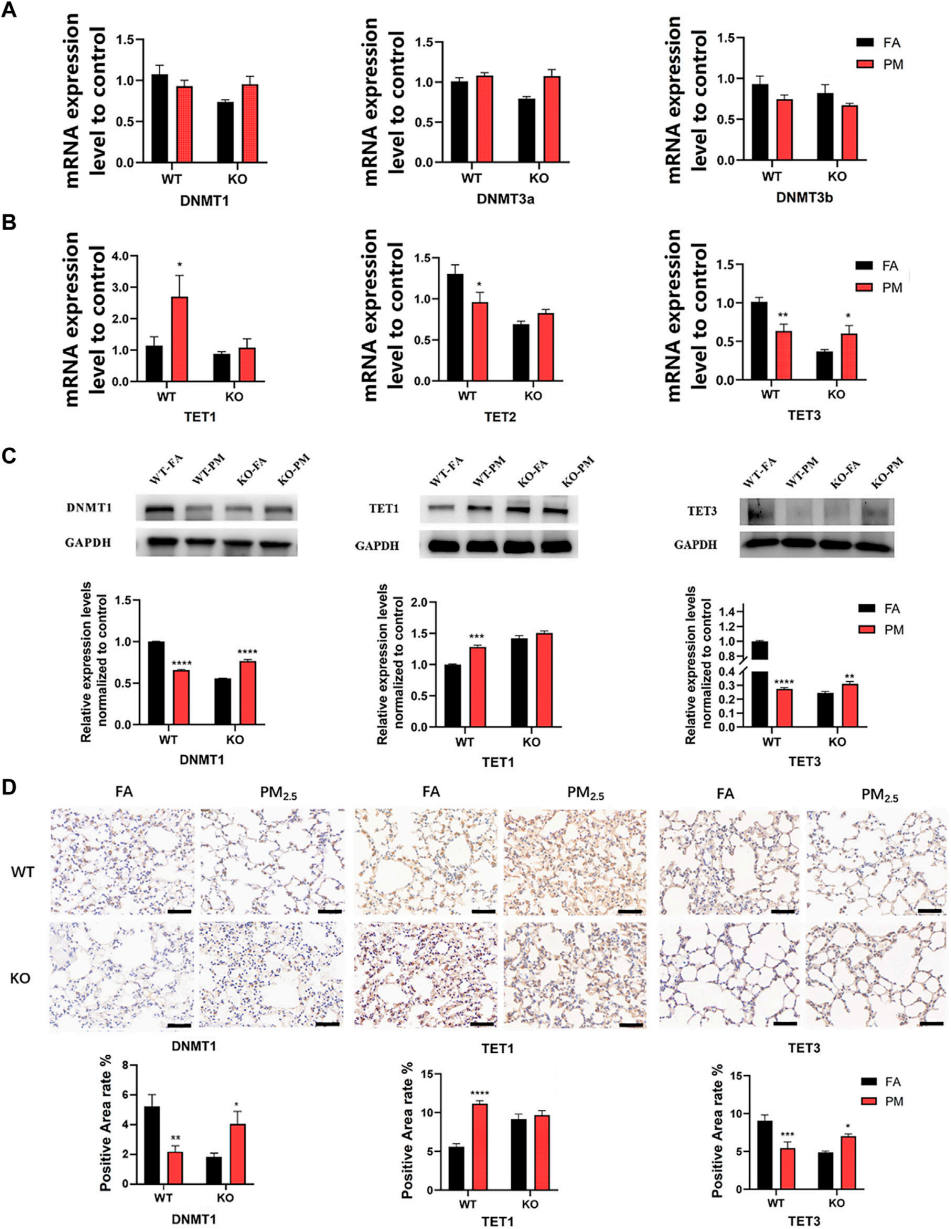
Expression of DNA methylation-related enzymes in four groups. **(A)** The DNA methyltransferase mRNA expression. *n* = 3 per group. **(B)** The DNA demethyltransferase mRNA expression levels. *n* = 3 per group. **(C)** WB analysis of protein levels of DNMT1, TET1, and TET3 expression. *n* = 3 in each group. **(D)** Immunohistochemical analysis of the positive area levels of DNMT1, TET1, and TET3. FA, filtered air; PM, fine particulate matter; WT, wild-type mice; KO, Nrf2^−/−^ mice. Scale bars are 50 μm, *n* = 3 per group. Error bars represent mean ± SEM, *p < 0.05,**p < 0.01,***p < 0.001,****p < 0.0001.

The level of DNA methylation is regulated by methylation-related enzymes. Therefore, we detected the expression of DNA methyltransferase (DNMT) and DNA demethyltransferase (TET). Our results indicated that TET1 mRNA was increased in WT mice after PM_2.5_ exposure, while TET2 and TET3 mRNA decreased significantly after exposure to PM_2.5_ (Figures 2A, B). However, only the TET3 mRNA level was significantly increased in Nrf2 knockout mice after PM_2.5_ exposure, and TDG mRNA expression of DNMTs, TET1, TET2, and TDG had no significant difference. (Figures 2A, B; Supplementary Figure S3). The protein and mRNA levels trend were similar, our data suggested that the protein levels of DNMT1 and TET3 were significantly decreased, while TET1 was increased in the WT mice following PM_2.5_ exposure. But the DNMT1 and TET3 protein levels in KO-PM mice were significantly higher than those in KO-FA mice, while TET1 showed no significant change (Figures 2C, D).

### 3.4 MassARRAY detection of methylation levels in CYP450 promoter

A single CpG site or multiple CpG sites constitute a methylation detection unit, and the methylation units in the detection region are detected. MassARRAY detection sequence *CYP2E1* contains 498 base pairs, 7 CpG sites are divided into 7 CpG units, *CYP1A1* contains 475 base pairs, 4 CpG sites are divided into 4 CpG units, *CYP2S1* contains 434 base pairs, including 15 CpG sites are divided into 12 CpG units.

**FIGURE 3:**
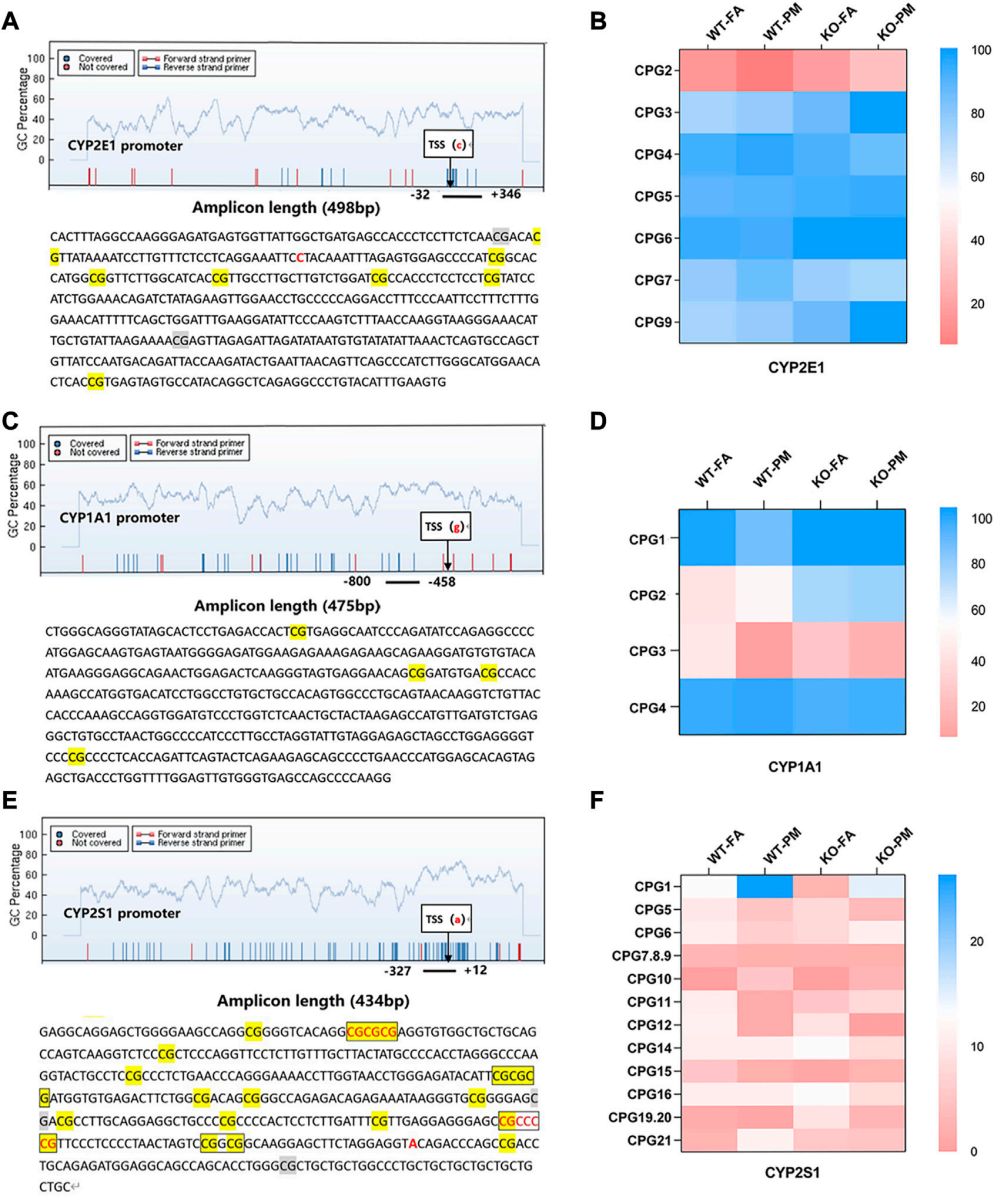
MassARRAY detects methylation levels in the CYP450 promoter; EpiTYPER was used for methylation sequence analysis of promoter selected sites. **(A)** Methylation units and sequences of the CYP2E1 promoter region. **(B)** Heat maps of the methylation levels of 7 CpG units of CYP2E1 in four groups. *n* = 3 per group. **(C)** Methylation units and sequences of the CYP1A1 promoter region. **(D)** Heat maps of the methylation levels of the 4 CpG units of CYP1A1. *n* = 3 per group. **(E)** Methylation units and sequences of the CYP2S1 promoter region. **(F)** Heat maps of the methylation levels of 12 CpG units of CYP2S1. n = 3 per group. FA, filtered air; PM, fine particulate matter. WT, wild-type mice; KO, Nrf2^−/−^ mice.

To investigate whether DNA methylation is the main factor affecting CYP450 expression, we detected the methylation levels of *CYP2E1, CYP2S1*, and *CYP1A1* promoter sites. In *CYP2E1* seven methylated CpG units, only the methylation level of CpG2 decreased in the WT group after PM_2.5_ exposure but increased in Nrf2^−/−^ group, and there was an opposite trend in CpG2 and CYP2E1 mRNA and protein levels; However, no similar trend was found in methylation levels of other CpG units and CYP2E1 expression (Figures 3A–C). Among all CpG units in the *CYP1A1* promoter, we found that compared with the control group, the methylation level of CpG3 in the WT and KO groups after PM_2.5_ exposure tended to decrease, which was contrary to the CYP1A1 mRNA level; However, there was no opposite trend between the remaining CpG units and CYP1A1 mRNA level (Figures 3C, D; Figure 1A). In the detected units of *CYP2S1*, only the methylation level of CpG1 increased after PM_2.5_ exposure in WT and Nrf2 knockout mice, and the trend of CpG1 was opposite to its mRNA levels. However, no similar trend was observed in the remaining CpG units (Figures 3E, F; Figure 1A). In conclusion, PM_2.5_ exposure in WT and Nrf2^−/−^ mice could affect the CpG unit methylation level of the CYP2E1, CYP1A1, and CYP2S1 enzymes, which might affect related gene expression.

### 3.5 Expression of global methylation levels in four groups

**FIGURE 4:**
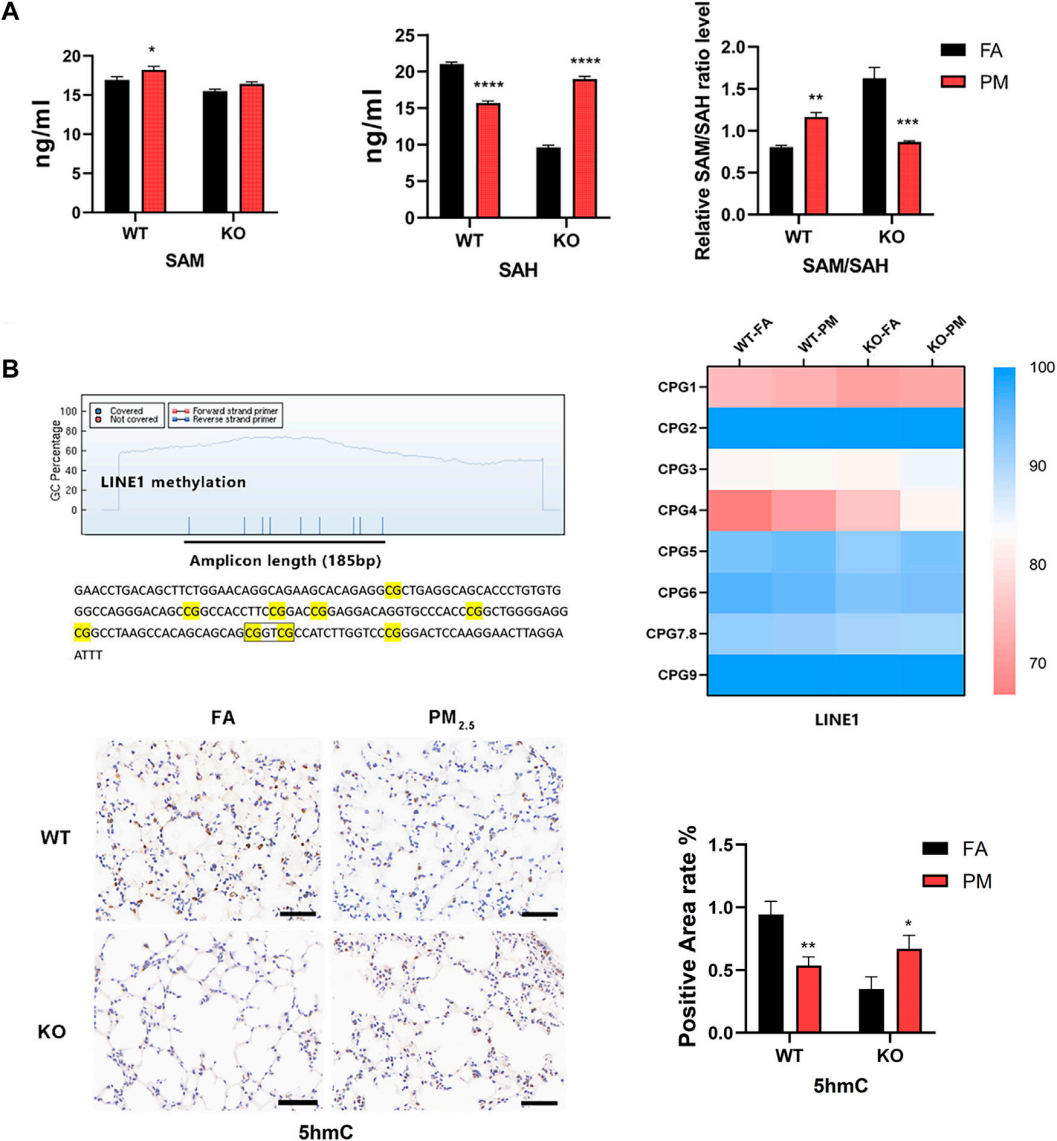
The global methylation-related indicators were at levels of the four groups. **(A)** Expression levels of SAM and SAH in lung tissue. *n* = 3 per group **(B)** Methylation units and sequences of the LINE1 region. MassARRAY assay for LINE1 methylation levels. *n* = 3 per group. The expression level of 5hmC in lung tissue was detected by immunohistochemistry and the positive area was calculated. *n* = 2 in per group. FA, filtered air; PM, fine particulate matter; WT, wild-type mice; KO, Nrf2^−/−^ mice. Scale bars are 50 μm. Data were mean ± SEM, *p < 0.05, **p < 0.01.

In DNA methylation analysis, *LINE1* methylation accounts for about 40%–50% of the whole genome (Choudhury et al., 2017), so we used MassARRAY to detect the methylation levels of *LINE1* was taken as the global methylation level, and the detection sequence consisted of 185 base pairs and 9 CpG sites were divided into eight CpG units. We found that CpG4 showed an increasing trend after PM_2.5_ exposure in WT and Nrf2 knockout mice, and the rest had no significant changes (Figure 4B), nevertheless, LINE1 mean methylation levels did not change significantly (Supplementary Figure S4). After PM_2.5_ exposure, the expression level of the WT group decreased at 5-hydroxymethylcytosine (5hmC), while the Nrf2 knockout mice increased (Figure 4B).

SAM (S-adenosylmethionine) is a raw material for DNA methylation and can affect DNA methylation, while SAH (S-adenosylhomocysteine) is a methyltransferase inhibitor, and the SAM/SAH ratio represents the potential for global methylation (Speckmann et al., 2017). In WT mice, the SAM expression level was elevated after PM_2.5_ exposure, while SAH was decreased, and the SAM/SAH was increased, so the potential for methylation was increased (Figure 4A). In Nrf2 knockout mice exposed to PM_2.5_, there was no obvious change in SAM, while SAH increased significantly, and SAM/SAH decreased significantly, so its methylation potential was significantly reduced (Figure 4A).

### 3.6 PM_2.5_ exposure induced ATR-dependent DNA damage repair in KO mice

**FIGURE 5:**
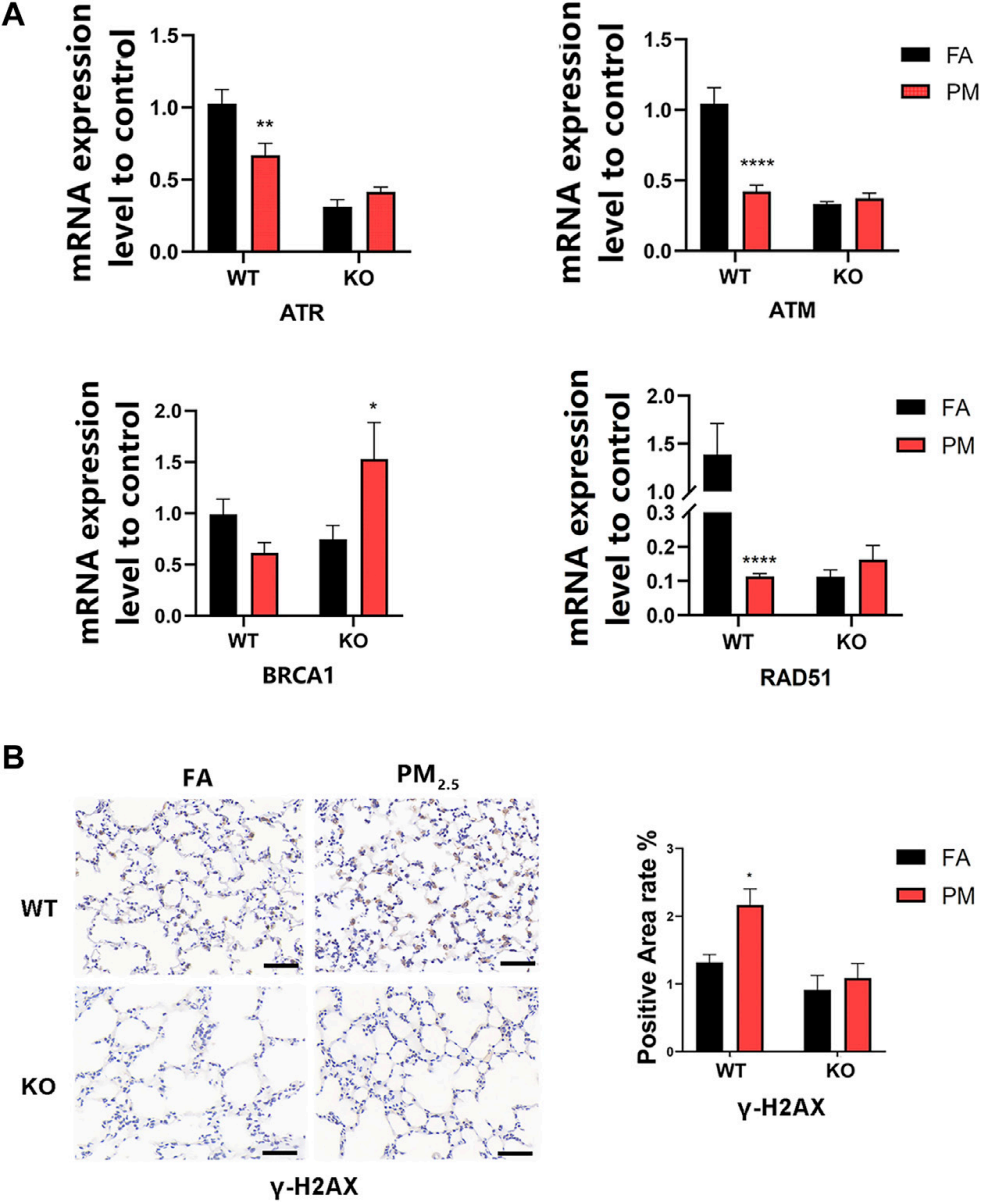
Effects of PM_2.5_ exposure on ATR pathway-related factors. **(A)**mRNA expression of ATR pathway-related indicators. *n* = 3 in each group. Data were mean ± SEM, *p < 0.05, **p < 0.01, ****p < 0.0001. **(B)** *γ*-H2AX expression level in mice lung tissue was detected by immunohistochemistry and the positive area was quantified by ImageJ. *n* = 2 per group. FA, filtered air; PM, fine particulate matter; WT, wild-type mice; KO, Nrf2–/– mice.

The study has found that Nrf2 could affect DNA damage repair by affecting the ataxia telangiectasia and Rad3-related protein (ATR) signaling pathway (Sun et al., 2020). To detect the effect of PM_2.5_ exposure on DNA damage repair, the mRNA levels of markers related to the ATR signaling pathway were measured. The *γ*-H2AX, an indicator of DNA damage, was increased in WT mice after PM_2.5_ exposure, but not in KO mice (Figure 5B). The mRNA levels of ATR pathway-related indicators ATR, ATM, RAD51, and BRCA1 decreased after the WT group was exposed to PM_2.5_, but increased after the KO-PM group (Figure 5A), suggesting that PM_2.5_ exposure induced ATR-dependent DNA damage repair in KO mice.

## 4 Discussion

Shijiazhuang City in Hebei Province experiences some of the most severe PM_2.5_ pollution in China. The city’s main sources of PM_2.5_ are industrial emissions, vehicle exhaust, and coal burning (Zhang et al., 2021). Because of its severe pollution, we selected this city for our study. We established a real-ambient exposure system in this area, which overcame the shortcomings of traditional exposure methods and better ensured that the temperature, air pressure, humidity, and all other conditions of the filtered air chamber (FA) and the PM_2.5_ exposure chamber (PM) were consistent, rendering the experimental results more comparable (Tang et al., 2022). We did not detect PM_2.5_ in the FA chamber, and the PM chamber could reflect the outside atmosphere. Studies using this system have been published in various journals (Li D. et al., 2019; Jiang et al., 2021).

Nrf2 is an important transcription factor for antioxidants. When stimulated by external environmental substances, the nuclear transcription of Nrf2 is increased, and the transcription of corresponding antioxidant genes is initiated (Liu et al., 2017). PM_2.5_ exposure increased the expression of Nrf2 and its antioxidant genes in mice lungs (Pardo et al., 2019), and Nrf2 protects the body from oxidative stress damage. A previous study found that PM_2.5_-induced oxidative stress leads to body damage, which is increased in Nrf2-deficient mice (Ge et al., 2020). However, other studies have shown that Nrf2 knockout did not exacerbate organ damage caused by PM_2.5_ exposure (Cui et al., 2020; Jiang et al., 2021).

Polycyclic aromatic hydrocarbons (PAHs) in PM_2.5_ can be metabolized and activated by CYP450, producing hazardous substances and causing lung injury (Martin et al., 2019). Our previous study showed that CYP2E1 is involved in lung injury caused by PM_2.5_. The injury caused by the increased expression of CYP2E1 may be related to the activation and metabolism of substances in PM_2.5_, thereby inducing endoplasmic reticulum stress (Ding et al., 2021). We showed that CYP2E1 expression was increased in WT-PM mice but decreased in KO-PM mice compared to controls, and thus CYP2E1 could play a key factor in the regulation of lung injury. However, the underlying mechanisms remain unclear.

The regulation of gene expression requires DNA methylation (Wagner et al., 2014). The hypermethylation or hypomethylation of gene promoters can silence or activate transcription, respectively (Baylin and Ohm, 2006). Much research has indicated that promoter DNA methylation regulates the expression of CYP450 enzymes (Suter et al., 2010; Jiménez-Garza et al., 2017; Takeda et al., 2021). It has been reported that DNA methylation may regulate CYP2E1 expression and enzyme activity in workers exposed to toluene (Jimenez-Garza et al., 2020). To clarify whether Nrf2 changed the gene expression of CYP450 enzyme (particularly CYP2E1) through altering DNA methylation following PM_2.5_ exposure, we investigated the methylation of the CYP2E1, CYP1A1, and CYP2S1 promoters, as well as DNA methylation-related indicators.

We found that the CpG2 methylation level in CYP2E1 was decreased in the WT-PM mice but increased in the KO-PM mice compared to the control. Interestingly, CYP2E1 mRNA and protein expression was increased in WT mice after PM_2.5_ exposure, but decreased in KO mice after PM_2.5_ exposure compared with control mice. This suggests that after PM_2.5_ exposure, CYP2E1 expression is increased in WT mice potentially *via* hypomethylation of the CpG2 unit, but is inhibited in KO mice potentially *via* CpG hypermethylation. Our results imply that Nrf2 could regulate CYP2E1 expression by affecting CpG2 methylation. We also found that the CpG3 methylation level in the CYP1A1 promoter decreased after exposure to PM_2.5_ in wild-type and Nrf2 knockout mice. However, PM_2.5_ exposure caused increased CYP1A1 mRNA expression in both wild-type and Nrf2 knockout mice. Therefore, we speculated that the CYP1A1 mRNA level might be increased by CpG3 hypomethylation in WT and KO mice following PM_2.5_ exposure. The methylation level of CpG1 in the CYP2S1 promoter region was increased in both WT and KO mice after PM_2.5_ exposure, which might be one of the reasons for the decreased mRNA expression of CYP2S1 in both WT and KO mice following PM_2.5_ exposure.

The major DNA modification was the methylation of cytosine (5 mC) (Li Z. et al., 2019). The DNA methylation process is mediated by DNMTs (Wu et al., 2007). According to the report that 5 mC is demethylated to 5hmC, 5 fC, and 5caC by TET oxidation (Ito et al., 2010). SAM/SAH ratio, DNMTs, and TETs are crucial to maintaining the homeostasis of DNA methylation in the body. Environmental pollution can affect single-carbon metabolic pathways through oxidative stress, leading to a decrease in methyl donor SAM or SAM/SAH levels, which may result in decreased DNA methylation (Wang et al., 2009). Air pollution may also affect the expression of DNMT and TET enzymes, which may result in decreased global methylation levels (Rider and Carlsten, 2019).

LINE1 methylation could represent global DNA methylation (Delgado-Cruzata et al., 2015). The previous study found that PM exposure can decrease LINE1 methylation in rat lungs (Ding et al., 2016; Guo et al., 2022). However, another study of boiler welders found no significant association between LINE1 methylation and occupational PM_2.5_ exposure (Kile et al., 2013). In this research, we found that the mean methylation level of LINE1 did not change significantly in the WT or KO group after PM_2.5_ exposure, indicating that exposure to PM_2.5_ might not affect the global methylation level. It was reported that 5hmC is a crucial product in enzyme-catalyzed active DNA demethylation (Hahn et al., 2013). We found that the 5hmC level was decreased in the lungs of WT mice following PM_2.5_ exposure, which was consistent with previous studies (de Oliveira et al., 2018), whereas in KO mice lungs increased. The SAM/SAH, an indicator of methylation potential, increased in WT mice after PM_2.5_ exposure, indicating an increase in methylation potential, but decreased in KO mice after PM_2.5_ exposure, indicating a decrease in methylation potential. We found that the SAM/SAH ratio was not associated with the methylation of LINE1. We speculated that the SAM/SAH ratio is too low to significantly affect the change in LINE1 methylation (Lee et al., 2017).

DNA methylation-related enzymes such as DNMTs and TETs can regulate DNA methylation and demethylation (Li and Zhang, 2014; Zhang et al., 2022). Many reports have shown that exposure to PM_2.5_ can generate oxidative stress, which inhibits the function of DNMTs, leading to global hypomethylation and even cancer (Franco et al., 2008). A population epidemiological study showed PM exposure resulted in a decrease in global DNA methylation and DNMT3B levels (Wang et al., 2020). According to another study, diesel particulate matter decreased TET1 expression in human bronchial epithelial cells and thereby a significant reduction in 5-hmC expression (Somineni et al., 2016). In the present research, we observed decreased expression of TET3 and DNMT1 when PM_2.5_ exposure in WT mice, which could be because PM_2.5_ exposure also reduces the enzyme activities of DNMT1 and TET3. However, there was no significant change in the DNA methylation level, which may be due to a decrease in the TET3 level leading to a decrease in the 5hmC level, causing an increase in 5 mC over time, thus maintaining DNA methylation0 homeostasis in the body (Rider and Carlsten, 2019). We speculated that the increase in the 5hmC level in KO mice exposed to PM_2.5_ was related to TETs; however, we found a significant increase only in TET3, which may be one reason for the increase in 5hmC. Our results suggest that Nrf2 may affect the global 5hmC level by regulating TET3 expression under exposure to PM_2.5_. However, compared with KO-FA mice, the global methylation level in KO-PM mice did not change significantly, whereas the expression of DNMT1 was significantly increased. This may be because the increased demethylation level in the body increases the expression of DNMT1 in the body *via* a feedback mechanism to maintain 5 mC homeostasis.

Overall, our results suggest that TET3 expression may affect the 5hmC level in WT and KO mice upon PM_2.5_ exposure. A previous study showed that ATR indirectly mediates the hydroxymethylation of 5mC to 5hmC by affecting the expression of TET3 in camptothecin-induced DNA damage in fibroblasts (Jiang et al., 2017), and recent studies have shown that Nrf2 regulates the ATR/Chk1 pathway is critical for radiation-induced DNA damage (Sun et al., 2020). We initially investigated whether Nrf2 affected TET3/5hmC levels *via* ATR following PM_2.5_ exposure. Interestingly, our results suggested that following exposure to PM_2.5_ in WT and KO mice, the expression of *ATR* and its pathway-related indicators was consistent with that of TET3 and 5hmC, implying that ATR expression may affect the expression of TET3 and 5hmC. In addition, after PM_2.5_ exposure, the changing trends in ATR, TET3, 5hmC, and other indicators in the WT group contrasted with those in the KO group, and we therefore speculated that Nrf2 mediates TET3/5hmC by affecting ATR after PM_2.5_ exposure.

In conclusion, in this research, a real-ambient exposure system was used to evaluate the influence of PM_2.5_ exposure on *CYP2E1*, *CYP1A1*, and *CYP2S1* expression possibly *via* CpG methylation in their promoters in WT and KO mice. Our previous study showed that Nrf2 could cause lung injury by inducing CYP2E1 after PM_2.5_ exposure (Ding et al., 2021). Here, we found that Nrf2 affected the CpG2 methylation level, which may be related to *CYP2E1* expression. Nrf2 might induce DNA demethylation *via* TET3 expression after PM_2.5_ exposure. These findings contribute to the understanding of epigenetic mechanisms after PM_2.5_ exposure and identify new mechanisms.

## Data Availability

The original contributions presented in the study are included in the article/Supplementary Material, further inquiries can be directed to the corresponding author.
