# Supplementary material for: Nrf2−/− regulated lung DNA demethylation and CYP2E1 DNA methylation under PM2.5 exposure
Source: Front Genet. 2023 Mar 20;14:1144903. doi: 10.3389/fgene.2023.1144903 (PMC10128193; doi:10.3389/fgene.2023.1144903)
Supplement: Supplementary file 1 [file DataSheet1.docx]

***Supplemental material***

**Contents**

**Table S1.** Primer pairs used in qRT-PCR.

**Table S2.** Primers used for MassARRAY

**Figure S1.** The expression level of Nrf2 was evaluated by western blot.

**Figure S2.** The level of oxidative stress in lung tissue decreased after PM_2.5_ exposure in Nrf2^-/-^ mice.

**Figure S3.** The mRNA expression level of TDG

**Figure S4.** The average methylation levels of LINE1

**Table S1**

| Gene |  | Forward Primer (5'-3') |  | Reverse Primer (5'-3') |
| --- | --- | --- | --- | --- |
| SOD1 |  | GCGTCATTCACTTCGAGCAGA |  | GGACCGCCATGTTTCTTAGAGT |
| SDO2 |  | AGACCTGCCTTACGACTATGG |  | CTCGGTGGCGTTGAGATTGTT |
| GSH-Px |  | CGCTCTTTACCTTCCTGCGGAA |  | AGTTCCAGGCAATGTCGTTGCG |
| GCLC |  | ACACCTGGATGATGCCAACGAG |  | CCTCCATTGGTCGGAACTCTAC |
| HO-1 |  | GATAGAGCGCAACAAGCAGAA |  | CAGTGAGGCCCATACCAGAAG |
| CYP2E1 |  | AGGCTGTCAAGGAGGTGCTACT |  | AAAACCTCCGCACGTCCTTCCA |
| CYP1A1 |  | ATCACAGACAGCCTCATTGAGC |  | AGATAGCAGTTGTGACTGTGTC |
| CYP2S1 |  | TGTCGTTGACGCCTTCCTGCTA |  | GCAAACAGCAGGTATGTGACCG |
| DNMT1 |  | AGGGACCATATCTGCAAGGAC |  | TATGGGCTATGACGCCATCTC |
| DNMT3a |  | GCACCAGGGAAAGATCATGTAC |  | AATGGAGAGGTCATTGCAGGG |
| DNMT3b |  | ACTTGGTGATTGGTGGAAGC |  | CCAGAAGAATGGACGGTTGTC |
| TET1 |  | CTCTTCCCACAGCCGATTCTCC |  | GCCGCTCATCTTCCACCTGAC |
| TET2 |  | CCCAAGATGGATAGTCATTTCA |  | CCGTGTAGCTGTAGATCGTGT |
| TET3  TDG  ATR  ATM  RAD51  BRCA1 |  | CATCAAGCAAGAGCCAATAGAC  GGGAACCTTGTGGCATTGCTTC  CTTATGCGGCTCAAGTCTGATT  TCTGTCCAGCAAAATCTCAAGG  AAGTTTTGGTCCACAGCCTATTT  GGGGAAAAGGTAGGTCCAAAC |  | TGCCACCTACACTGTTAGTCCT  GCAAACGACTGTGCCATCCACT  TCTGAACTCCAAGAGCGTCTAC  CCCATGTAACAATAGCAGCCAA  CGGTGCATAAGCAACAGCC  CTGCTTCAGCATTTGACTCGT |
| β-actin |  | CTACCTCATGAAGATCCTGACC |  | CACAGCTTCTCTTTGATGTCAC |

**Table S2**

| Gene |  | | Forward Primer (5'-3') | |  | | Reverse Primer (5'-3') | |
| --- | --- | --- | --- | --- | --- | --- | --- | --- |
| CYP2E1 | | aggaagagag TATTTTAGGTTAAGGGAGATGAGTGG | |  | |  | | cagtaatacgactcactatagggagaaggct CACTTCAAATATACAAAACCTCTAAACC |
| CYP1A1 | | aggaagagag TTGGGTAGGGTATAGTATTTTTGAGATT | |  | |  | | cagtaatacgactcactatagggagaaggct CCTTAAAACTAACTCACCCACAACT |
| CYP2S1  LINE1 | | aggaagagag GAGGTAGGAGTTGGGGAAGTTAG  aggaagagag GAATTTGATAGTTTTTGGAATAGGT | |  | |  | | cagtaatacgactcactatagggagaaggct ACAACAACAACAACAACAACAAAAC  cagtaatacgactcactatagggagaaggct AAATTCCTAAATTCCTTAAAATCCC |


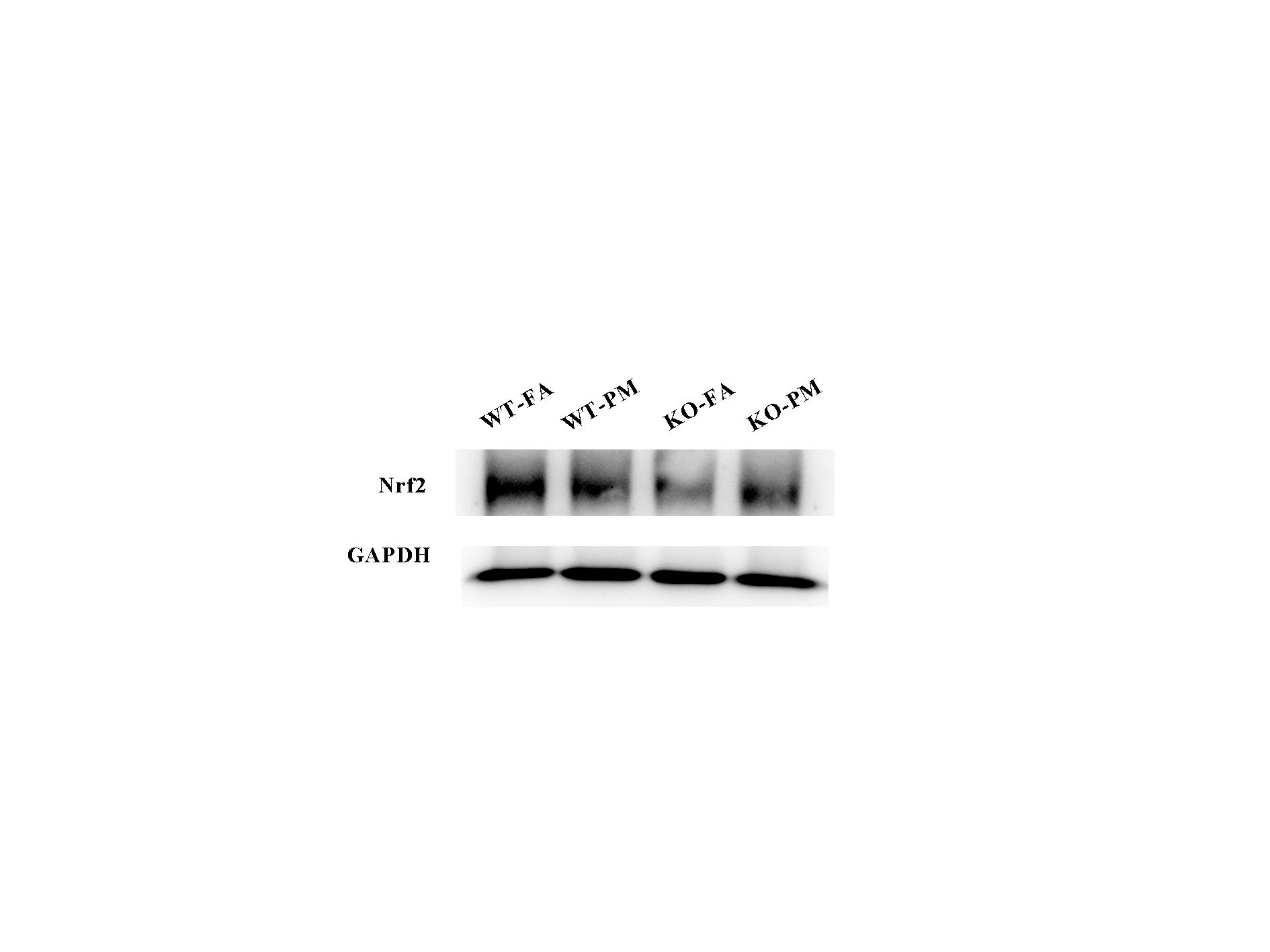

**Figure S1,** Nrf2 protein expression was measured by WB and quantified by ImageJ (NIH, United States)


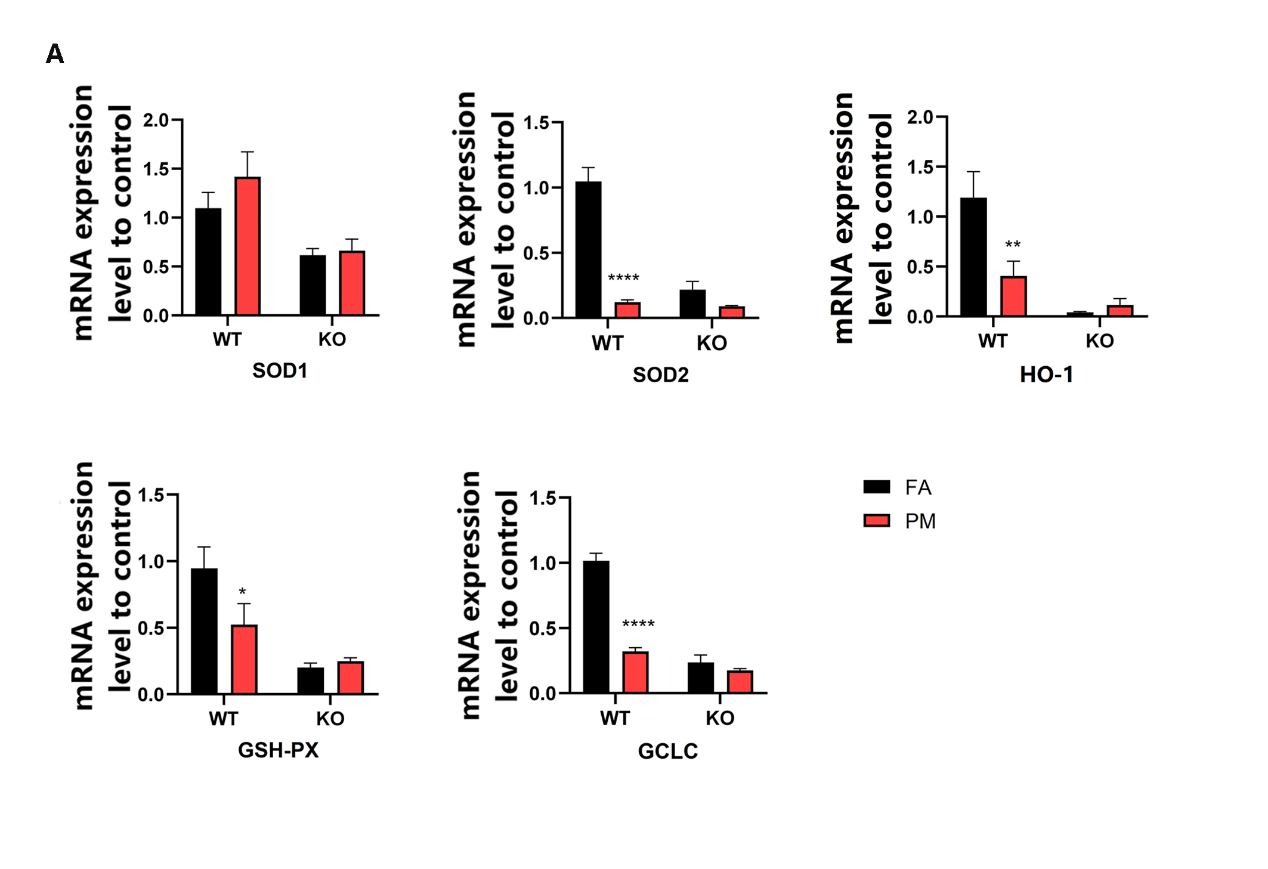


**Figure S2.** The level of oxidative stress in lung tissue decreased after PM2.5 exposure in Nrf2-/- mice. The expression of four groups of antioxidant indexes was detected by qPCR. n=3 per group. FA, filtered air; PM, fine particulate matter; WT, wild-type mice; KO, Nrf2-/- mice. Data are expressed as the mean ± SEM, *p< 0.05, ****p< 0.0001.

**
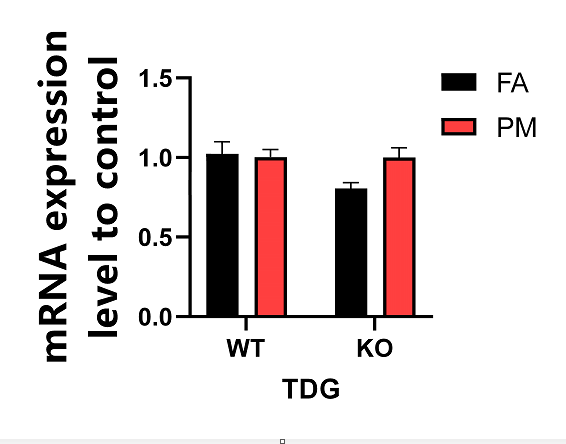
**

**Figure S3.** The mRNA expression level of TDG. N=3 per group. FA, filtered air; PM, fine particles. WT, wild-type mice; KO, Nrf2^-/-^ mice. Data are expressed as the mean ± SEM, *p< 0.05.


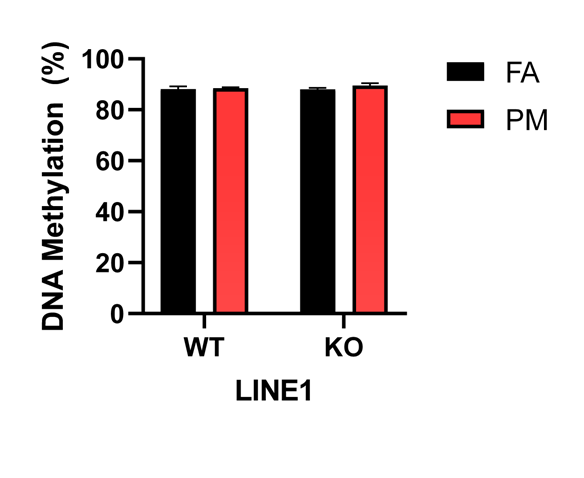


**Figure S4** LINE1 methylation levels were measured using MassARRAY, N=3 per group. Data are expressed as the mean ± SEM, *p< 0.05.

Jianguoyun supplement data link:

https://www.jianguoyun.com/p/DXF7ld8Q1p2pCxiqyPMEIAA
